# Proteomic Characterization of Bottlenose Dolphin (*Tursiops truncatus*) Urine

**DOI:** 10.1021/acs.jproteome.5c00718

**Published:** 2025-12-13

**Authors:** Kelly N. Cusick, Alison Bland, Nicole I. Stacy, Wayne E. McFee, Ryan Takeshita, Randall S. Wells, Cynthia R. Smith, Lori Schwacke, Michael G. Janech

**Affiliations:** 1 Department of Biology, Grice Marine Laboratory, 2343 College of Charleston, Charleston, South Carolina 29412, United States; 2 Hollings Marine Laboratory, Charleston, South Carolina 29412, United States; 3 Department of Large Animal Clinical Sciences, College of Veterinary Medicine, 3463 University of Florida, Gainesville, Florida 32611, United States; 4 National Centers for Coastal Ocean Science, 7202 National Oceanic and Atmospheric Administration, NOAA Charleston, Charleston, South Carolina 29412, United States; 5 338774 National Marine Mammal Foundation, San Diego, California 92106, United States; 6 Brookfield Zoo Chicago’s Sarasota Dolphin Research Program, c/o Mote Marine Laboratory, Sarasota, Florida 34236, United States; 7 Marine Mammal Commission, Bethesda, Maryland 20814, United States

**Keywords:** acute kidney injury, antimicrobial proteins, tandem mass spectrometry, marine mammal

## Abstract

Urinary proteins offer multifaceted insights into tissue repair, dysfunction, and renal health, with significant implications for both human and veterinary medicine. However, marine mammal medicine lacks comprehensive studies on urine protein composition. This study aimed to describe the urine proteome of wild common bottlenose dolphins (*Tursiops truncatus*) at two Gulf of Mexico sites (Sarasota Bay, FL, and Barataria Bay, LA) and to compare urine proteins by sex. Ten urine samples (Barataria Bay, LA: *N* = 6; Sarasota Bay, FL: *N* = 4) were analyzed by nano LC-MS/MS. Peptide spectral matching identified 1872 protein families across all individuals (FDR < 0.01). Cystatin 11 was notably present in males (median rank abundance: 8.1%) and absent in females (median: 0.0%), with semen contamination elevating protein diversity in male urine. Two putative antimicrobial proteins, cathelicidin and lysozyme, accounted for 2.66% of the urine proteome, suggesting an innate immune defense mechanism. In total, 27 proteins that are recognized as acute kidney injury markers in humans, and 12 putative stone formation proteins were detected in dolphin urine. This research provides a reference database of urinary proteins that can be used to develop advanced methods for investigating dolphin renal health. Data are available via ProteomeXchange with identifier PXD054283.

## Graphical abstract

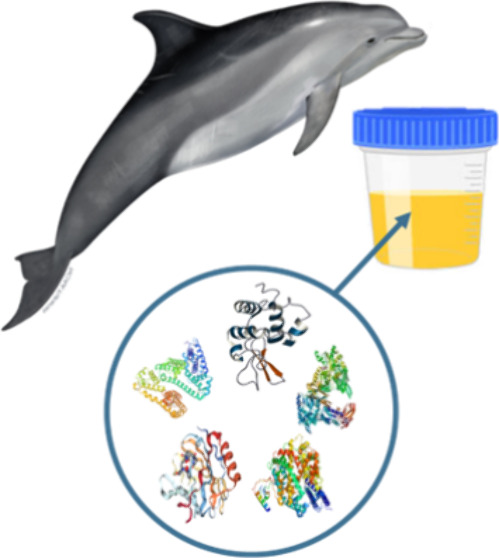

## Introduction

Advances in urinary proteomics for human medicine have facilitated its application in diagnostics, primarily through the use of tandem mass spectrometry as a tool for identification and quantification of urinary proteins related to renal health.^1–5^ The presence and composition of proteins in urine can provide detailed insights into various physiological processes within the kidney under both normal and pathophysiological conditions.^1,4,5^ Several proteins have emerged as candidate markers of renal injury in veterinary medicine, including neutrophil gelatinase-associated lipocalin (NGAL; approved name: lipocalin 2; approved symbol: LCN2) and liver-type fatty acid binding protein (LFABP; approved name: fatty acid binding protein 1; approved symbol: FABP1), both of which are elevated in the urine following renal injury in dogs.^6,7^ The early detection of specific urinary proteins can help veterinarians identify evidence of renal dysfunction, allowing for more prompt and targeted intervention. However, despite these advances, similar diagnostic tools have yet to be investigated in cetaceans because the urinary proteome of cetaceans and marine mammals in general have not yet been described with the exception of California sea lions.^8^ Studies to determine whether the aforementioned proteins indicative of renal injury also behave as markers of injury in nonmodel organisms (e.g., marine mammals) cannot be conducted until it is known that a similar marker protein exists in the sample of a species of interest. Once identified, a study can be conducted to investigate changes in a specific urinary protein marker to tissue injury or disease. This highlights the gap in translating known protein markers of humans and domesticated species to nonmodel organisms.

The application of urine protein chemistry in dolphins remains an underexplored area of cetacean health research. Although bottlenose dolphins are commonly studied, standard tools used to evaluate kidney function in humans have not been widely adapted or validated for this species. Methods such as dipstick tests often produce inconsistent results,^9,10^ and without established reference interval data considering partitioning factors, it can be difficult to interpret protein concentrations of clinical concern.^11^ These limitations hinder efficient diagnostic applications to assess renal health in bottlenose dolphins beyond serum biomarkers.

Aside from health assessment applications, urine proteomics can provide insights into adaptations among different mammals. For instance, studies on the urine of California sea lions (*Zalophus californianus*) revealed that antimicrobial proteins, such as resistin and lysozyme, were present in higher relative abundance than in humans and domesticated dogs, indicating that their excretory system may have evolved to combat pathogens by upregulating the innate immune system.^8^ Similarly, high concentrations of lysozyme in the urine of Bactrian camels (*Camelus bactrianus*) and giraffes (*Giraffa camelopardalis*) further suggest that over-representation of innate immune proteins in the urine is characteristic of wild mammals, although very few studies have been conducted.^12,13^ These findings highlight the potential of urine proteomics to uncover innate immune strategies across species. It is unclear whether bottlenose dolphins also excrete urine with over-representation of innate immune effector proteins similar to those found in California sea lions and some terrestrial artiodactyls.

The current study characterized the urine proteome of free-ranging bottlenose dolphins sampled from two Gulf of Mexico sites (Sarasota Bay, FL and Barataria Bay, LA), with the goal to establish a database of urinary proteins that can serve as a reference for determining which urine proteins are excreted by bottlenose dolphins. This database can be further studied as potential biomarkers of disease for diagnostic purposes and as a resource for comparative studies across mammals. In addition, we compared our urine proteome results for bottlenose dolphin, a species that are part of the Artiodactyla order, with previously published data for California sea lions in order to examine similarities and differences between marine mammals of differing taxonomic orders. We observed and discussed urine proteins in bottlenose dolphins that may be of clinical interest to wildlife veterinarians due to their use as biomarkers of acute kidney injury (AKI) in both human and domestic animals. We also identified urine proteins associated with kidney stone formation or inhibition due to the prevalence of kidney stones in bottlenose dolphins under managed care.^10,14–18^

## Methods

### Sample Collection

In 2023, ten urine samples were collected from free-ranging bottlenose dolphins as part of the catch-and-release health assessments in the Gulf of Mexico, USA. Free-ranging bottlenose dolphins were temporarily caught, sampled, and released under well-tested, standardized protocols approved through NOAA/National Marine Fisheries Service (NMFS) Marine Mammal Protection Act (MMPA) Scientific Research Permits and annual IACUC reviews, as previously described.^19,20^ In May 2023, paired urine and serum samples were collected from bottlenose dolphins captured and released in Sarasota Bay, FL (n = 4) by the Brookfield Zoo Chicago’s Sarasota Dolphin Research Program (SDRP) under the NMFS Scientific Research Permit No. 26622. Samples collected from the Sarasota Bay sampling effort are indicated with the letter “F” in the sample name. In June 2023, the National Marine Mammal Foundation (NMMF) collected paired urine and serum samples from bottlenose dolphins captured and released in Barataria Bay, LA (n = 6) under NMFS Scientific Research Permit No. 24359. Samples collected from the Barataria Bay sampling effort are indicated with the letter “Y” in the sample name.

Prior to urethral catheterization, the restrained bottlenose dolphin was placed into a sling and lifted onto the processing vessel (Figure 1). The bottlenose dolphin was positioned in the right lateral recumbency, and the genital slit was irrigated twice with sterile water (McKesson, product number: 520119) using a 10 mL syringe. Subsequently, approximately 125 mL of sterile water was poured over the external area of the genital slit after irrigation. The type of urinary catheter used for Sarasota Bay bottlenose dolphins was either 8 Fr x 22” or 5 Fr x 16” (Kendall) urinary catheter, which was chosen at the discretion of the attending veterinarian. For the Barataria Bay bottlenose dolphins, 8 Fr x 42” nasogastric feeding tube (JorVet) was used for urethral catheterization. The tip of the urinary catheter was covered in sterile surgical lubricant (Surgilube) and inserted into the urethral opening of the male bottlenose dolphin as shown in (Figure 1). Urine samples of 1 to 20 mL were collected in 50 mL falcon conical tubes and immediately placed on ice in an onboard cooler for 2 to 6 h. Within 8 h of collection, the urine samples were centrifuged at 1,500xg for 10 min at ambient temperature to isolate the cell-free urine supernatant. Supernatant was transferred into 2 to 5 mL cryovials and stored at −80 °C.

**Figure 1:**
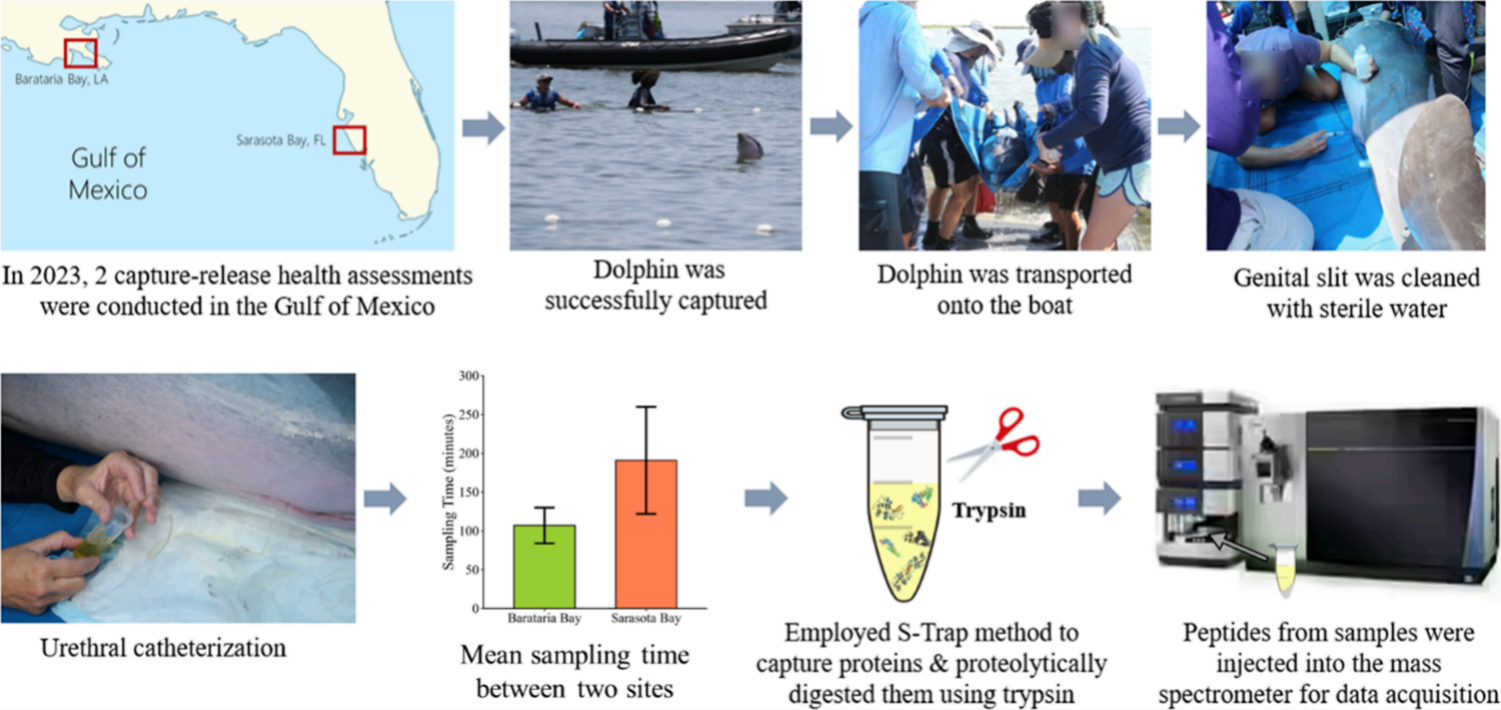
Summary of the experimental workflow, from animal capture to sample preparation, and nanoLC-MS/MS. Mean sampling time from capture to urine collection differed significantly (*p* < 0.05) between Barataria Bay (191 ± 69 min; green bar) and Sarasota Bay (107 ± 23 min; orange bar) sites. Error bars indicate standard deviation. Photos taken by the National Marine Mammal Foundation under NMFS Scientific Research Permit no. 24359. Photographs courtesy of Dr. Ryan Takeshita. Copyright 2023.

Dry reagent dipstick strip measurements and urine sediment analyses of fresh urine samples were performed at the University of Florida for the Sarasota Bay bottlenose dolphins using Siemens Multistix 10 SG reagent strips (Siemens Medical Solutions USA, Inc. Malvern, PA, USA). For the Barataria Bay bottlenose dolphins, samples were thawed at Cornell’s Animal Health Diagnostic Center for dry reagent dipstick strip measurements and urine sediment analyses using Siemens Clinitek Urinalysis analyzer and Siemens Multistix 10 dipsticks (Siemens Medical Solutions USA, Inc., Malvern, PA, USA). Dry reagent dipstick strips were used to record protein, glucose, ketones, bilirubin, heme, and the value of specific gravity and pH. The urine sediment analysis included evaluation for presence and number of white blood cells, red blood cells, bacteria, epithelial cells, sperm, casts, and crystals in the urine. Frozen urine samples were thawed at room temperature (22 °C) and vortexed for 15 s for urine osmolality measurements, as determined by the VAPRO (model 5600, ELITech Group, Logan, Utah, USA) vapor pressure osmometer. Total protein was determined using the pyrogallol method (QuanTtest Red Total Protein Assay System, Quantimetrix Corp) and polyacrylamide gel electrophoresis using 1.0 mm x 10 wells NuPAGE 4–12% Bis-Tris gels.

As part of the health assessment of bottlenose dolphins, blood samples were drawn from the periarterial rete on the underside of the tail fluke above the water using a 19-gauge x 3/4” Scalp Vein Butterfly Winged Infusion set (Merit Pharmaceutical) into the 10 mL serum separator tubes. Serum chemistry analyses of all samples were performed on the Cobas C501 (Roche Diagnostics Corporation, Indianapolis, IN, USA) at Cornell’s Animal Health Diagnostic Center.

### Protein Digestion

Mass spectrometry grade reagents were used in all preparation steps described below. Urine samples were thawed quickly in a water bath at 37 °C and vortexed for 10 min at room temperature. Urine proteins were digested according to protocols established for the S-Trap method (Protofi). In 1.5 mL Lo-Bind microcentrifuge tubes (Eppendorf), a volume of urine supernatant equal to 50 μg total protein was solubilized in an equal volume of 2x Lysis Buffer (10% SDS, 100 μM TEAB, pH 7.55) and vortexed for 10 min at room temperature. The mixture was then centrifuged at 13,000xg for 8 min at room temperature (22 °C). Samples were reduced using a volume of 90 mM dithiothreitol (DTT) to achieve a final concentration of 10 mM DTT, heated at 60 °C for 30 min in a heat block, and cooled to room temperature (22 °C). Samples were quickly centrifuged to collect condensed water at the lid. Alkylation was performed by adding a volume of 200 mM chloroacetamide (CAA) to reach a final concentration of 20 mM CAA, followed by a vortex of 10 s and incubation at room temperature (22 °C) in the dark for 30 min. To each sample, 12% phosphoric acid was added at a 1:10 v:v ratio to achieve a final concentration of 1.2% phosphoric acid. Samples were then diluted with the S-Trap binding buffer (100 mM TEAB in 90% methanol, pH 7.52) in a 1:7 v:v ratio of sample to S-Trap binding buffer. The diluted samples were transferred to S-Trap digestion mini columns (Protofi) attached to a vacuum manifold (model DOA-P704-AA, GAST Manufacturing, Inc., Benton Harbor, MI, USA) and vacuum was applied at −0.22 BAR or −700 kPa (mmHg) until the solution was pulled through the column. The captured proteins were washed 6 times with S-Trap binding buffer on the vacuum manifold. Proteins trapped on the S-Trap digestion mini columns were digested by adding 5 μg of Pierce trypsin protease in digestion buffer (50 mM ammonium bicarbonate in water, pH 8.5), following the manufacturer’s protocol. The S-Trap digestion mini columns containing protease were then incubated at 47 °C for 2 h in an Isotemp incubator (model 203FS, Thermo Fisher Scientific, Dubuque, IA, USA). Proteins were eluted sequentially using 80 μL of digestion buffer, followed by centrifugation at 4,000xg for 60 s at 22 °C. Sequential elution was performed with 80 μL of 0.2% formic acid followed by 80 μL of 50% acetonitrile containing 0.2% formic acid, with centrifugation at 4,000xg for 60 s at 22 °C between each addition. All eluates were pooled in 1.5 mL Lo-Bind tube and stored at −80 °C. Samples were concentrated using a SpeedVac (model SC110A, Savant Instruments Inc., Holbrook, NY, USA) for approximately 2 h or until about 20 μL remained in the Lo-bind tubes.

Tryptic peptides were resuspended in 200 μL of 0.2% formic acid. Solid-phase cartridges (Affisep, C18-cartridges, product number: Spin-C18.T1.96) were activated with 200 μL acetonitrile and equilibrated with 200 μL of 0.2% acetonitrile/formic acid (80%/20% ratio), followed by 200 μL of 0.2% formic acid prior to the addition of peptides. Each addition was accompanied by centrifugation at 1,200xg for 2 min at 22 °C. The cartridges were subsequently loaded with acidified samples (200 μL of 0.2% formic acid), followed by centrifugation at 1,200xg for 2 min at 22 °C and a wash with 200 μL of 0.2% formic acid. The cartridges were then eluted twice with an elution buffer containing 50% acetonitrile (volume fraction) in 0.2% formic acid (volume fraction) with a centrifugation at 1,200xg for 2 min at 22 °C after each elution. The eluates were then freeze-dried using a SpeedVac for 3 h. The resulting tryptic peptides were resuspended in 50 μL of 0.1% formic acid to a final concentration of 0.1 μg/μL prior to injection and data acquisition via tandem mass spectrometry (nanoLC-MS/MS).

### Bottom-up Proteomics–Mass Spectrometry (nanoLC-MS/MS)

A total of 500 ng of tryptic peptides resuspended in 0.1% formic acid (volume fraction) were analyzed using parallelized dual-trap single-column liquid chromatography (DTSC) approach^21^ on the UltiMate 3000 Nano LC coupled to a Fusion Lumos Orbitrap mass spectrometer (Thermo Fisher Scientific). The UltiMate 3000 Nano LC setup was equipped with two 10-port valves, two trap columns, and one analytical column. A volume equivalent to 500 ng of peptide was injected onto one of the two dual-trap single-columns, Thermo C18 PepMap 100 μ-precolumn trap cartridges (300 μm id x 5 mm length). A constant loading pump flow rate of 15 μL/min was maintained with 2% acetonitrile (volume fraction) and 0.05% trifluoroacetic acid (volume fraction). The parallel DTSC method performs both trapping and elution simultaneously, allowing one sample to be loaded and trapped while the previous sample is being eluted. During the elution step, peptides were eluted from the trap column and separated on the analytical column [Thermo Acclaim PepMap RSLC 2 μm C18 column (75 μm id x 15 cm length), at 40 °C] before entering the mass spectrometer at a flow rate of 300 nL/min. Mobile Phase A was 0.1% formic acid in water (volume fraction), and Mobile Phase B was 80% acetonitrile, 20% water, and 0.08% formic acid (volume fractions). The gradient began at 9% Mobile Phase B and ramped up to 30%B over 51 min, then to 50%B from 51 to 63 min, followed by an increase to 98%B from 63 to 64.5 min, held until 69 min, and dropped back to 9% Mobile Phase B by 69.1 min, and held for an additional 5.9 min for a total of 75 min.

As the above gradient was running, the loading pump operated in reverse flow over the trap for 5 min. During this time, 25 μL of a 50% methanol and 50% acetonitrile (volume fraction) mixture was injected twice, followed by an injection of 25 μL of 0.1% formic acid (volume fraction) in water. Next, the flow direction was switched forward, and the sample was loaded. At 73 min, the valve switched again, briefly reversing the flow across the secondary trap to remove precipitates and desalting back to waste. This was followed by forward loading of the sample to be eluted into the analytical column connected to the mass spectrometer.

The Fusion Lumos was operated with 60 s of dynamic exclusion (with 10 ppm error) in positive ion mode with a 30% RF lens and data-dependent mode (topN, 3s cycle time). The scan mass range was set to a 375 to 1500 mass-to-charge (*m*/*z*) ratio with the resolution set at 60,000. The maximum injection time of 50 ms was allowed with a full scan ion target value set at approximately 4.0 × 10^5^. The peptides were specified with an intensity threshold of 5.0 × 10^4^ for precursor selection, including charge states 2 to 7 for monoisotopic peak determination. Fragment ion data was collected at a resolution of 30,000 for data-dependent peptide fragmentation.

### Data Processing

Raw files were converted to peak lists in.mgf format using MSConvert (v3.0.18205; available at https://github.com/phnmnl/container-pwiz). The following databases were specified for the search: *T. truncatus;* NCBI RefSeq mTurTru.1.mat.Y; GCF_011762595.1, containing 55,741 sequences and the common Repository of Adventitious Proteins database (cRAP; 2012.01.01; Global Proteome Machine), containing 107 sequences. Protein databases from Refseq were chosen to maximize peptide identification sensitivity by using a database that included a more comprehensive coverage of protein encoding genes, including computationally predicted genes. The larger size of the RefSeq database reflects an annotation strategy for nonmodel organisms, where extensive manual curation is lacking. An error-tolerant search was performed using Mascot (v2.8.2) on the merged .mgf files to identify predominant fixed and variable post translational modifications, essential for the subsequent 2-step search strategy described below. Following the error tolerant search and selection of variable modifications, the search parameters were configured as follows: trypsin was specified as the enzyme with up to three mis-cleavages allowed. Carbamidomethyl (C) was set as a fixed modification, while deamidated (NQ) and oxidation (M) were set as variable modifications. Precursor mass tolerance (MS_1_) was set to 10 ppm and product mass tolerance (MS_2_) to 30 ppm, generated in MetaMorpheus (v1.0.5; available at https://github.com/smith-chem-wisc/MetaMorpheus). The instrument type was specified as ESI-FTICR, and the decoy setting in Mascot was used to estimate the local false discovery rate (FDR). Subsequently, a second step in the search pass was conducted on the unassigned list of spectra from the initial search for each urine sample using semitrypsin as the enzyme. Variable modifications included dehydroalanine (C), carbamidomethyl (C), deamidation (NQ), and oxidation (M), with no fixed modification specified. Mass tolerances of 10 ppm for MS_1_ and 30 ppm for MS_2_ were applied to this secondary search as well. Results from both first and second searches were combined for each urine sample and peptides were scored using the Scaffold (v5.1.1, Proteome Software, Portland, OR, USA) local FDR algorithm with a protein and peptide FDR thresholds set to 1% and 0.1%, respectively, for positive identifications with a minimum of one peptide. Following the exclusion of decoy and cRAP hits, exponentially modified protein abundance index (emPAI)^22^ was exported from Scaffold to Rstudio (v2024.04.2–764). Raw data, search results, and databases have been deposited to the ProteomeXchange Consortium via the PRIDE partner repository^23^ with the data set identifier PXD054283 and 10.6019/PXD054283.

### Comparison to California Sea Lion Proteome

Raw data files (.raw) and search results of the California sea lion urine proteome were acquired from the ProteomeXchange Consortium via the PRIDE partner repository, with the data set identifier PXD009019 and DOI: 10.6019/PXD009019. Nondiseased California sea lions, categorized as “Control”, were selected for direct comparisons with bottlenose dolphins. The.mgf files were imported into Mascot using parameters detailed in Neely et al. (2018), with searches conducted against the following specified databases: *Z. californianus;* NCBI RefSeq mZalCal1.pri.v2; GCA_009762305.2, containing 61,078 sequences, and the common Repository of Adventitious Proteins database (cRAP; 2012.01.01; Global Proteome Machine), containing 107 sequences. Search results were imported into Scaffold with a set protein and peptide FDR threshold of 1% for positive identifications. Following the exclusion of cRAP and decoy hits, the resulting normalized emPAI^22^ data was exported to Rstudio (v2024.04.2–764).

For comprehensive functional annotations of the bottlenose dolphin and California sea lion urine proteomes, protein families were aligned with their human equivalents using the HUGO Gene Nomenclature Committee (HGNC) gene symbols using a python script (https://github.com/neely/ChatGPT/tree/main/addGeneSymbol2RefSeq). In instances where a gene symbol was unavailable, NCBI’s protein–protein BLAST (Basic Local Alignment Search Tool) was utilized for human sequence similarity searches for manual assignment of gene symbols. Assignments were based on top similarity results showing at least 65% identity and an expectation value (E-value) of ≤ 1e-5 against the human genome, identified by accession number beginning with NP (*Homo sapiens*, taxid: 9606). In g:Profiler (https://biit.cs.ut.ee/gprofiler/gost), the gene symbols were used to query the Human Protein Atlas under the protein database to determine localized protein enrichment in tissues^24^. Statistical parameters within g:Profiler were configured to consider “only annotated genes”, significance threshold was set to “Benjamini-Hochberg FDR”, user threshold was set to “0.05”, and Numeric IDs were treated as “ENTREZGENE_ACC”. Homologous proteins based on matching gene symbols identified in the bottlenose dolphins and California sea lions for ranking of abundance comparison. The presence and absence of identified proteins in the semen proteome of bottlenose dolphins^25^ was determined in the urine proteome of bottlenose dolphins based on matching gene symbols. Manual inspection of gene symbols in the bottlenose dolphin urine proteome was completed to select urine proteins implicated as biomarkers for AKI in humans and in kidney stone formation in both human and veterinary research via literature search.

The molecular weights of proteins in the bottlenose dolphin reference proteome were calculated by summing the monoisotopic masses of all amino acids in each protein sequence in the current *T. truncatus* database described above using Python (v3.12.3) (https://github.com/specht/proteomics-knowledge-base/blob/master/amino-acids.csv). To determine the distribution of the molecular weights for bottlenose dolphin plasma proteins, raw data from the previously published bottlenose dolphin plasma proteome^26^ were acquired and searched using the current *T. truncatus* database. Search modifications were applied according to those described by Miller et al. (2017), with the data set identifier PXD005435. The distribution of serum protein molecular weights was derived from the Scaffold file (.sf3) of the bottlenose dolphin serum proteome, as provided by Sobolesky et al. (2016), with the data set identifier PXD005435 and DOI: 10.6019/PXD003425. Scaffold was used to predict the molecular weights of proteins identified in the urine, plasma, and serum proteomes based on their amino acid sequences from the current *T. truncatus* database.

### Data Analysis

Ages were determined from regular systematic photographic identification surveys for individuals sighted since birth or estimated by pectoral flipper radiography for those not sighted since birth.^27,28^ To facilitate age class comparisons across sites, corresponding length thresholds for the age categories were derived from previously published growth curves of another Northern Gulf of Mexico bottlenose dolphin stock, Mississippi Sound.^29^ Subsequently, urine samples were categorized into the following age classes: juvenile (≤5 years), subadult (>5 and <10 years), and adult (≥10 years).

Normally distributed continuous clinical data between groups with equal variances were compared using the student’s *t* test, while the Welch’s *t* test was employed for groups with unequal variances. Non–normally distributed clinical data were analyzed using the Mann–Whitney U test. Differences between demographic data were determined using independent chi-square tests. Twenty-four h renal protein excretion was not measured. All values are reported as mean ± standard deviation (SD).

Urine protein compositions were ranked based on percent molar ratio using emPAI (eq 1).^22^

$$\mathrm{Molar}\;\mathrm{ratio}(\%)=\frac{emPAI}{\sum (emPAI)}\times 100$$ 1

For differential analysis of proteomic data, a two-sided Mann–Whitney U test was used for pairwise comparisons between sexes and species because 87% of the proteomic data were not normally distributed as determined by the Shapiro-Wilk test, respectively. To control for false discoveries, Mann–Whitney U tests were adjusted using the Benjamini-Hochberg method.^30^ Principal component analysis (PCA) was employed to examine multivariate relationships among sample distributions, considering factors such as sex and site using weighted spectral counts (wSC).

The molecular weight distribution for the number of identified proteins was calculated as the proportion of the number of proteins within a specified molecular weight range in bins of 10 kDa for each proteome. A chi-squared test of independence was conducted to evaluate the difference between proteome type and molecular weight distribution. The AKI-associated proteins were ranked based on mean molar ratio (%) in the bottlenose dolphin urine. Statistical significance was accepted at *p* < 0.05.

## Results

### Serum and Urine Chemistry

Urine and serum samples (N = 10) were analyzed from Sarasota Bay, FL (3 females, 1 male) and Barataria Bay, LA (2 females, 4 males) bottlenose dolphins (Table 1 and Table S1). All bottlenose dolphins included in this study were considered clinically normal at the time of sampling, with no observable injuries or signs of illness based on veterinary examinations and serum chemistry data. Mean sampling time from capture to urine sample collection was higher in the Sarasota Bay group compared to the Barataria Bay group (191 ± 69 [107–273] minutes vs 107 ± 23 [52–107] minutes, respectively, *p* < 0.05, Student’s *t* test; Figure 1). Samples collected from bottlenose dolphins at the Sarasota Bay site exhibited 1.6% lower serum sodium compared to the Barataria Bay group (152.5 ± 1.29 mEq/L vs 155 ± 1.26 mEq/L, respectively, *p* < 0.05, Student’s *t* test). Serum chloride was 2.8% lower in bottlenose dolphins from Sarasota Bay compared with those in Barataria Bay site (114.3 ± 1.26 mEq/L vs 117.5 ± 2.43 mEq/L, respectively, *p* < 0.05, Student’s *t* test). Serum sodium and chloride were within the reference range for free-ranging bottlenose dolphins (Table 1).^31^ Values of other serum chemistry analytes (including BUN, SCr, calcium, phosphorus, and potassium) were not significantly different between sites. The Sarasota Bay bottlenose dolphins displayed lower urine specific gravity (USG) compared to the Barataria Bay group (1.014 ± 0.005 vs 1.040 ± 0.003, respectively, *p* < 0.001, Student’s *t* test), whereas urine osmolality did not differ between sites. There was no difference in the mean urine total protein concentration between bottlenose dolphins from Sarasota Bay those from Barataria Bay (10.25 ± 9.25 mg/dL vs 55.33 ± 50.75 mg/dL, respectively, *p* > 0.05, Student’s *t* test).

**Table 1:** Urine and Serum Chemistry Analytes of Bottlenose Dolphins in This Study, Accompanied by Pertinent Characteristics such as Sex and Age^a^

	Barataria Bay (*N* = 6)	Sarasota Bay (*N* = 4)	*p*-value	statistical value	reference data
sex (male/female)	4/2	1/3	0.52	*X* ^2^ = 0.42	
age class					
juvenile (≤5 years)	0	2	0.13	*X* ^2^ = 4.05	
subadult (>5 and <10 years)	1	0			
adult (≥10 years)	5	2			
serum creatinine (SCr), mg/dL					
mean ± SD	1.22 ± 0.26	1.03 ± 0.25	0.28	*t* = 1.15	0.9–1.9
range	0.90 −1.70	0.70–1.30			
BUN, mg/dL					
mean ± SD	59 ± 13	59 ± 4	1.0	*t* = 0	42–76
range	41–80	54–63			
BUN/Cr					
mean ± SD	51 ± 15	63 ± 20	0.38	*t* = −0.92	25.7–82.4
range	24–67	42–90			
serum sodium, mEq/L					
mean ± SD	155 ± 1	153 ± 1	0.016	*t* = 3.04	152–160
range	154–157	151–154			
serum chloride, mEq/L					
mean ± SD	118 ± 2	114 ± 1	0.041	*t* = 2.43	107–124
range	114–121	113–116			
serum calcium, mg/dL					
mean ± SD	9.1 ± 0.2	9.2 ± 0.5	0.66	*t* = −0.46	8.5–10.1
range	8.9–9.4	8.5–9.6			
serum phosphorus, mg/dL					
mean ± SD	4.7 ± 0.3	4.9 ± 0.3	0.47	*t* = −0.76	3.3–6.8
range	4.4–5.1	4.5–5.2			
serum potassium, mEq/L					
mean ± SD	4.2 ± 0.4	3.9 ± 0.2	0.16	*U* = 19	3.2–4.5
range	3.7–4.7	3.7–4.0			
urine specific gravity					
mean ± SD	1.040 ± 0.003	1.014 ± 0.005	5.36 × 10^–6^	*t* = 10.6	
range	1.036–1.044	1.010–1.020			
urine osmolality (mOsm/kg)					
mean ± SD	1320 ± 197	1103 ± 179.4	0.12	*t* = 1.76	
range	1037–1607	943–1332			
urine protein (mg/dL)					
mean ± SD	55.3 ± 50.8	10.3 ± 9.3	0.067	*U* = 21	
range	12–122	4–24			
urine protein > 30 mg/dL	3	0	0.324	*X* ^2^ = 0.32	

a Abbreviations: BUN = blood urea nitrogen, SCr = serum creatinine. Statistical probabilities were calculated using Chi-squared tests (*X* ^*2*^), Student’s t-tests (*t*), or Mann–Whitney U tests (*U*). Data included are mean ± standard deviation (SD) and a range from minimum to maximum measured values. Serum reference data of free-ranging bottlenose dolphins are obtained from Schwacke et al.

### Urine Proteome

A total of 1,872 urine protein families were identified experiment-wide, excluding contaminant and decoy proteins (Table S2). For Sarasota Bay bottlenose dolphins, identified proteins represented 1,522 unique protein families (mean: 851 ± 168 per individual). Proteins identified from bottlenose dolphins in the Barataria Bay site represented 1,414 unique protein families (mean: 592 ± 293 per individual). There was no difference between the number of proteins identified between sites (*p* > 0.05, Mann–Whitney U test). A total of 1,653 unique protein families were identified in the male bottlenose dolphins (mean: 776 ± 212 per individual), while a total of 1,207 unique protein families (mean: 615 ± 268 per individual) were identified in the female group. There was no difference between the number of proteins identified between sexes (*p* > 0.05, Mann–Whitney U test).

Using an arbitrary inclusion threshold of 0.5%, the topmost 30 urine proteins constituted two-thirds of the total protein abundance (Figure 2). When proteins were ranked by mean molar ratio, albumin (ALB) emerged as the most abundant protein, representing an average of 12.9% of the urine protein composition, followed by lymphocyte antigen 6 family member D (LYD6) and cystatin 11 (CST11). Uromodulin (UMOD) ranked 22nd among the most abundant urine proteins, accounting for 0.8% of the bottlenose dolphin urine protein composition (Figure 2). CST11, which was not previously seen in the urine proteomes of humans, dogs, or California sea lions,^1,8,32,33^ was identified among the abundant urine proteins in bottlenose dolphins, but this protein is found in only male bottlenose dolphins (mean rank: 3). Several proteins within the topmost 30 abundant proteins of the bottlenose dolphin urine proteome ranked considerably higher compared to the relative abundance reported for the human urine proteome based on an intensity-based absolute quantification (iBAQ) algorithm.^32^ These proteins include cathelicidin (CAMP; dolphin rank: 9 vs human rank: 1,450), NHERF family PDZ scaffold protein 1 (NHERF1; dolphin rank: 27 vs human rank: 1,001), guanylate cyclase activator 2B (GUCA2B; dolphin rank: 10 vs human rank: 1,072), NPC intracellular cholesterol transporter 2 (NPC2; dolphin rank: 16 vs human rank: 384), lysozyme (LYZ; dolphin rank: 21 vs human rank: 524), cystatin A (CSTA; dolphin rank: 25 vs human rank: 752), and superoxide dismutase 1 (SOD1; dolphin rank: 29 vs human rank: 340) (Table S2).^32^

**Figure 2:**
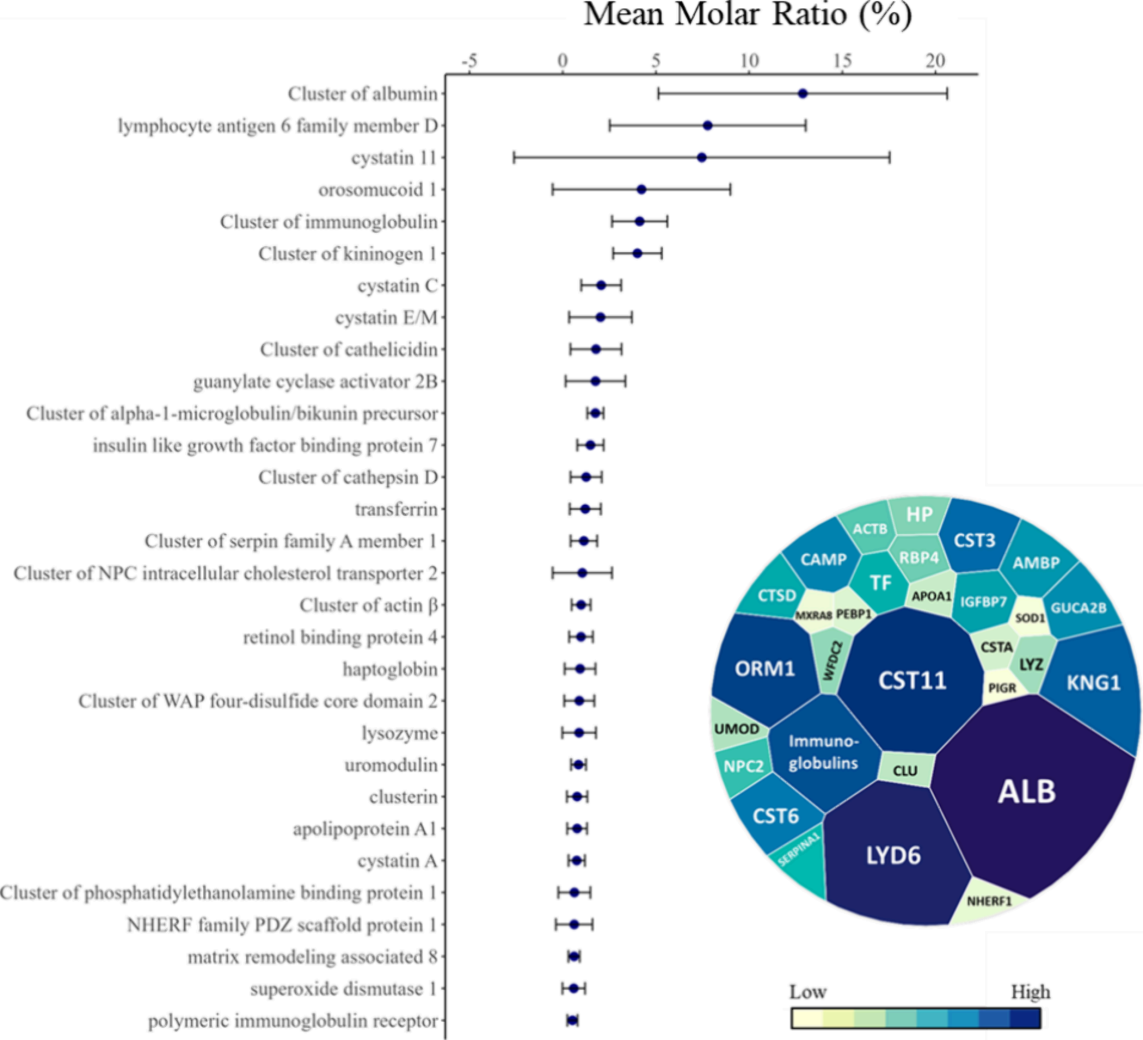
Mean molar ratio (%) of the top 30 abundant proteins in the urine of ten bottlenose dolphins, determined using normalized emPAI values. The cutoff was arbitrarily selected to include proteins that represented greater than 0.5% of the urine proteome and these 30 proteins comprised 2/3 of the molar ratio from the urine. There were 30 proteins that comprised 2/3 of the molar ratio from the urine. The topmost abundant protein, albumin represented 12.9% of the bottlenose dolphin urine protein composition. The bars represent the 95% confidence intervals. The accompanying voronoi treemap illustrates urine protein abundance with low abundance shown in yellow and high abundance represented in blue. Protein names were converted to HGNC-approved names along with their corresponding gene symbols.

### Gene Ontology (GO) Terms & Human Protein Atlas

The HGNC-approved symbols of bottlenose dolphin urine proteins were cross-referenced with the Human Protein Atlas using g:Profiler to determine enrichment within nephron segments **(** Figure 3).^24^ This analysis revealed that the majority of the identified proteins were enriched in kidney tissues (59.9%), followed by a high abundance of proteins observed for the proximal tubules (11.5%), distal tubules (10.8%), and collecting ducts (9.6%) compared to the Bowman’s capsule (4.6%) (*p* < 0.05; Figure 3). However, urine proteins were not significantly abundant in the other two categories associated with kidneys such as cells in the glomeruli and cells in the tubules. Since the loop of Henle was not included as one of the nephron categories in Human Protein Atlas analysis, it is possible that there are proteins enriched in both thick and thin limbs in the loop of Henle.

**Figure 3:**
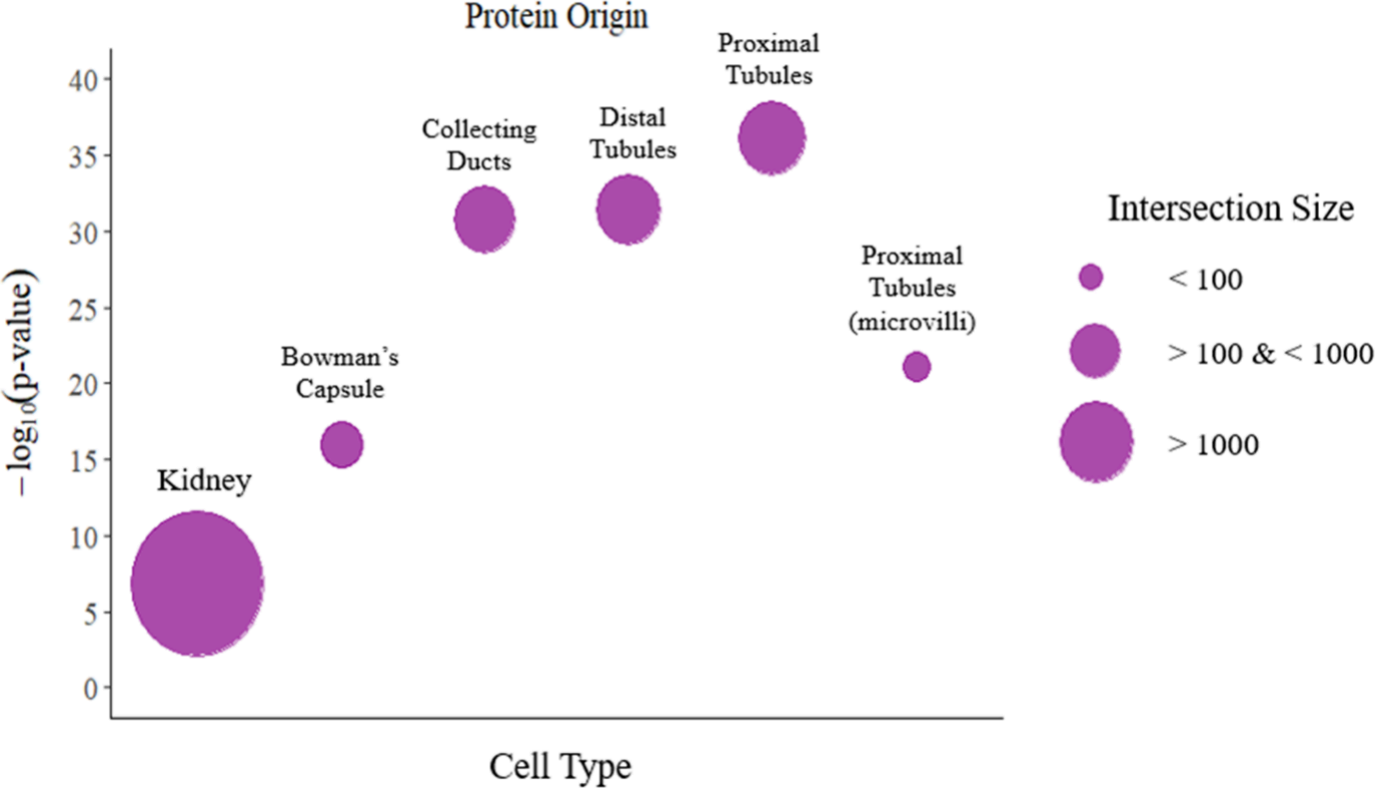
Abundance of urine proteins identified within the kidney segments (cell type) of bottlenose dolphins. Protein enrichment analysis was set to a low level of protein abundance within the kidney using the Human Protein Atlas tool in g:Profiler (*p* < 0.05). The size intersection corresponds to the abundance.

### Molecular Weight Distribution

The molecular weight distribution of identified proteins in the bottlenose dolphin urine proteome differed significantly from those of the reference, serum, and plasma proteomes (χ^2^ = 233.1, df = 60, *p* < 0.05, chi-squared test, Figure 4). The mean molecular weight of the bottlenose dolphin urine proteome was 64.9 ± 109.9 kDa (SD), which was lower than that of the reference (75.5 ± 69.0 kDa), serum (79.4 ± 75.7 kDa), and plasma (74.2 ± 68.9 kDa) proteomes. Proteins in the 10–39 kDa range were significantly overrepresented in the bottlenose dolphin urine proteome compared to the other proteomes, whereas, proteins in the 60–69 kDa, 80–99 kDa, 110–139 kDa, 150–159 kDa, 170–179 kDa, and >200 kDa ranges were underrepresented. Molecular weight bins were defined in 10 kDa increments (e.g., 30–39 kDa includes proteins from 30 to 39 kDa).

**Figure 4:**
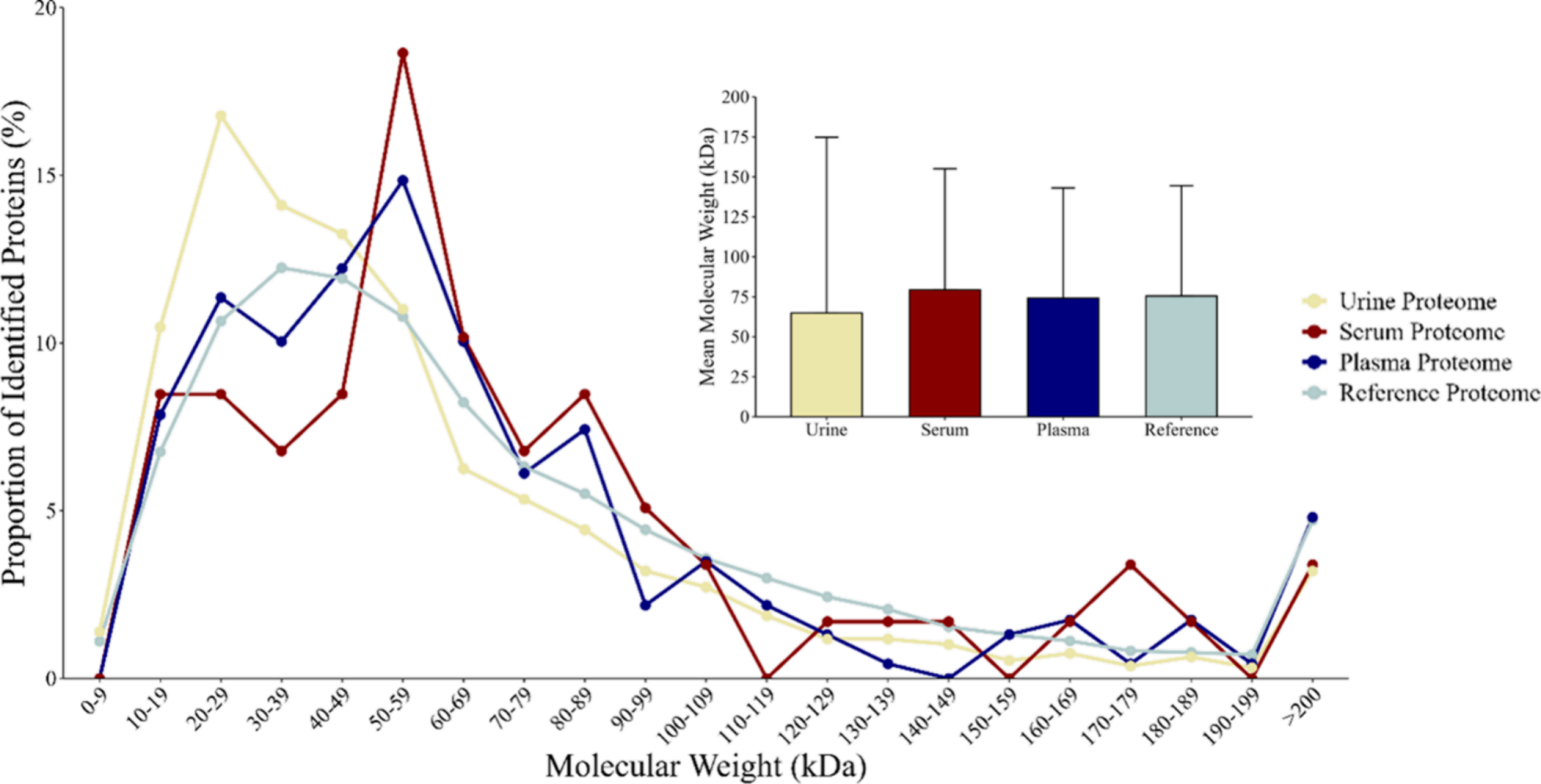
Bottlenose dolphin urinary proteome is comprised of lower molecular weight proteins compared to the plasma or serum proteome, but not exclusively low molecular weight proteins. Relative frequency (%) of molecular weight (kDa) distribution of the reference bottlenose dolphin proteome (green lines; *N* = 55,741), urine proteome (yellow lines; *N* = 1872), plasma proteome (blue lines; *N* = 229), and serum proteome (red lines; *N* = 59). Relative frequency was calculated as the percentage of proteins falling within each 10 kDa bin, relative to the total number of proteins in the respective proteome. The molecular weight distribution differed significantly among the four proteomes, as evidenced by chi-squared test of independence (χ^2^ = 233.1, df = 60, *p* < 0.05). Mean molecular weight of proteins in urine (yellow bar), serum (red bar), plasma (blue bar) and reference proteomes (green bar), with upward standard deviation error bars.

There was no significant difference (*p* > 0.05, chi-squared test) in the predicted molecular weight distribution based on the number of identified proteins in bins of 0 kDa to 59 kDa, 60 kDa to 69 kDa, and greater than 69 kDa between the bottlenose dolphin urine proteome and the California sea lion urine proteome (Figure 5). In the bottlenose dolphin urine proteome, 1,237 of 1,872 proteins (66.1%) were predicted to weigh less than 60 kDa, 125 proteins (6.7%) were predicted to weigh 60–69 kDa, and 510 proteins (27.2%) were predicted to weigh greater than 69 kDa. In the California sea lion urine proteome, 1,058 of 1,583 proteins (66.8%) were predicted to weigh less than 60 kDa, 110 proteins (6.9%) were predicted to weigh 60–69 kDa, and 415 proteins (26.2%) were predicted to weigh greater than 69 kDa.

**Figure 5:**
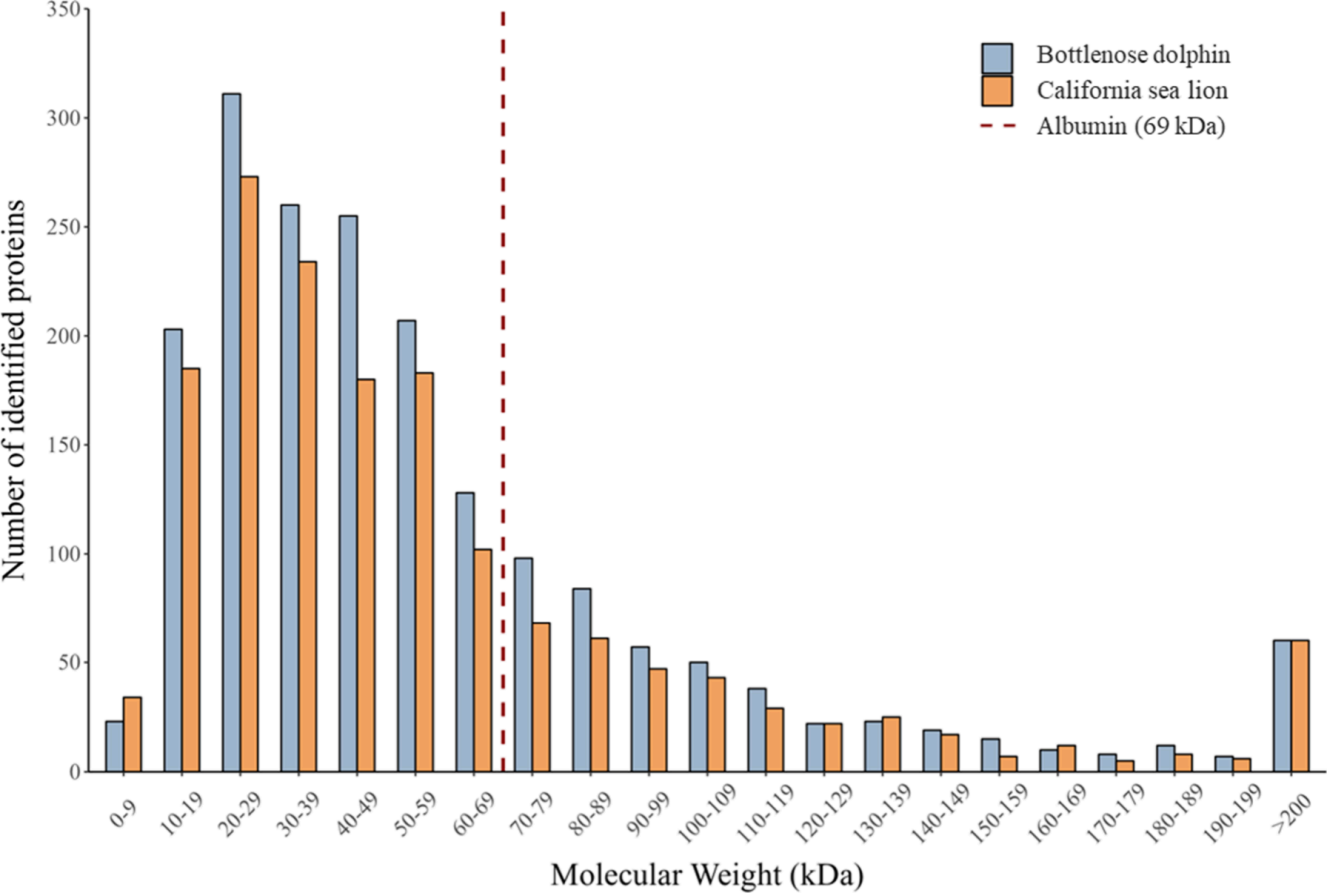
Comparison of hypothetical protein size of urinary proteins between bottlenose dolphins and sea lions displays similarities between both marine mammals. Molecular weight (kDa) distribution of the number of identified proteins within each 10 kDa bins for all proteins plotted across both bottlenose dolphins (blue; *N* = 1872) and California sea lions (orange; *N* = 1583) groups. Urine protein molecular weight distributions in bins of 0 to 59, 60 to 69, and greater than 69 kDa were not considered different between groups as indicated by the chi-squared test of independence (*X* ^2^ = 0.51, df = 2, *p* > 0.05). The red dashed line represents albumin weighing at 69 kDa.

### Comparison of Urinary Protein Composition between Sexes and Sites

The distribution of urine proteins (wSC) across all individuals was stratified into two groups based on sex and site. Principal component analysis (PCA) was conducted instead of pairwise statistics for observational purposes due to the low number of individuals. Principal component 1 and principal component 2 explained 24.6% and 17.9% of the total variance, respectively (Figure 6). No discernible clustering of the groups by sex or site was observed within the 95% confidence intervals. Two outliers appeared in both PCA plots, corresponding to male bottlenose dolphin urine samples containing sperm postcentrifugation: one from Sarasota Bay (F292) and one from Barataria Bay (YR0), as confirmed via urine sediment review by light microscopy (Table S1).

**Figure 6:**
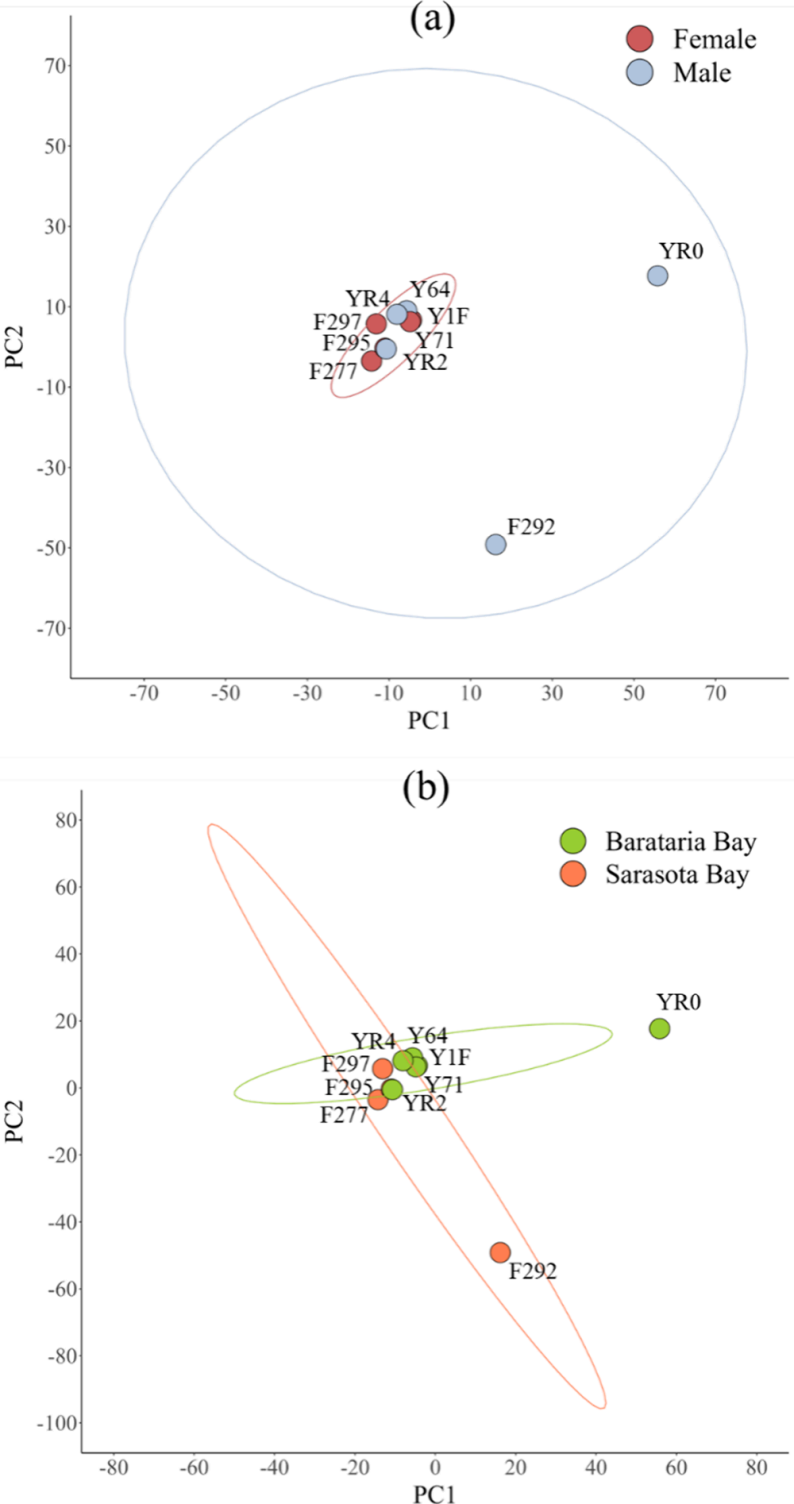
Scatterplots of the principal component 1 (PC1; 24.6%) and principal component 2 (PC2; 17.9%) scores were derived from wSC of urine proteins from all bottlenose dolphins denoted by their field identification code. Grouping was based on (a) sex, with female (red) and male (blue), and (b) site, with Barataria Bay (green) and Sarasota Bay (orange). Ellipses denote the 95% confidence intervals. No distinct clustering of groups was observed for both sex and site.

When molar protein ratios were examined among the top 30 proteins between males and females, there were no significant differences (*p*_*adj*_ > 0.05, Mann–Whitney U test, Benjamini-Hochberg corrected) with the exception of CST11, with a mean rank of 3 (Figure 2). CST11 was present in four of five males (median: 8.1%) but was absent in females (median: 0.0%; *p*_*adj*_ > 0.05, Mann–Whitney U test, Benjamini-Hochberg corrected; Figure 7). Given that CST11 is primarily associated with male reproductive tissues, including sperm and testis,^34^ the bottlenose dolphin semen proteome data set was juxtaposed with the female and male bottlenose dolphin urine proteome data sets (Figure 8).^25^ The analysis revealed that 427 of 607 identified protein families (70.3%) in the semen proteome were also present in the bottlenose dolphin urine samples (Figure 8). The semen proteome encompassed two biological fluids: seminal plasma and spermatozoa. The two urine samples containing sperm (YR0 and F292; Table S1) heavily contributed to the number of identified protein families shared between the male urine proteome and seminal plasma and spermatozoa. Specifically, 55 out of 66 of the seminal plasma protein families (83.3%), all 118 of the sperm protein families (100%), and 29 out of 37 of the protein families shared between seminal plasma and sperm (78.4%) were exclusively found in these two male urine samples. On average, the number of identified protein families in these two male urine samples containing sperm (984 ± 58 per individual) was 1.5-fold greater than the number of identified protein families in male urine samples without sperm (638 ± 127 per individual, *p* < 0.05, Student’s *t* test).

**Figure 7:**
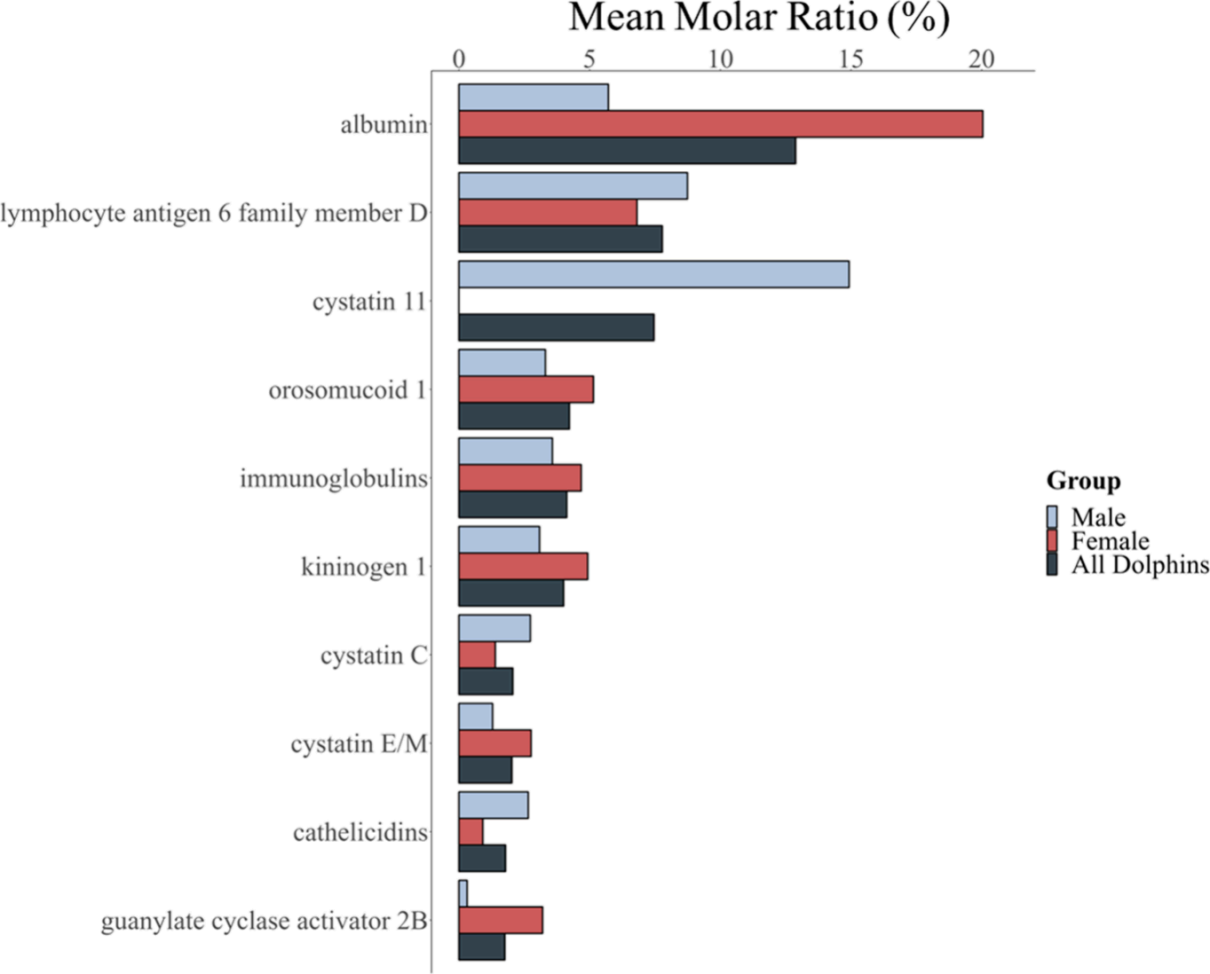
Distribution of the most abundant urine proteins in bottlenose dolphins by sex shows reveals cystatin-11 as a male urinary protein. Mean molar ratio (%) of the top 10 abundant proteins in the urine of male (blue), female (red), and bottlenose dolphins (black) (*N* = 10) displayed on the *y*-axis for comparison across groups. No significant differences in urine protein composition were observed between groups, as determined by Mann–Whitney U tests with Benjamini–Hochberg adjustments (*p*_*adj*_ > 0.05). Protein names were converted to HGNC-approved names.

**Figure 8:**
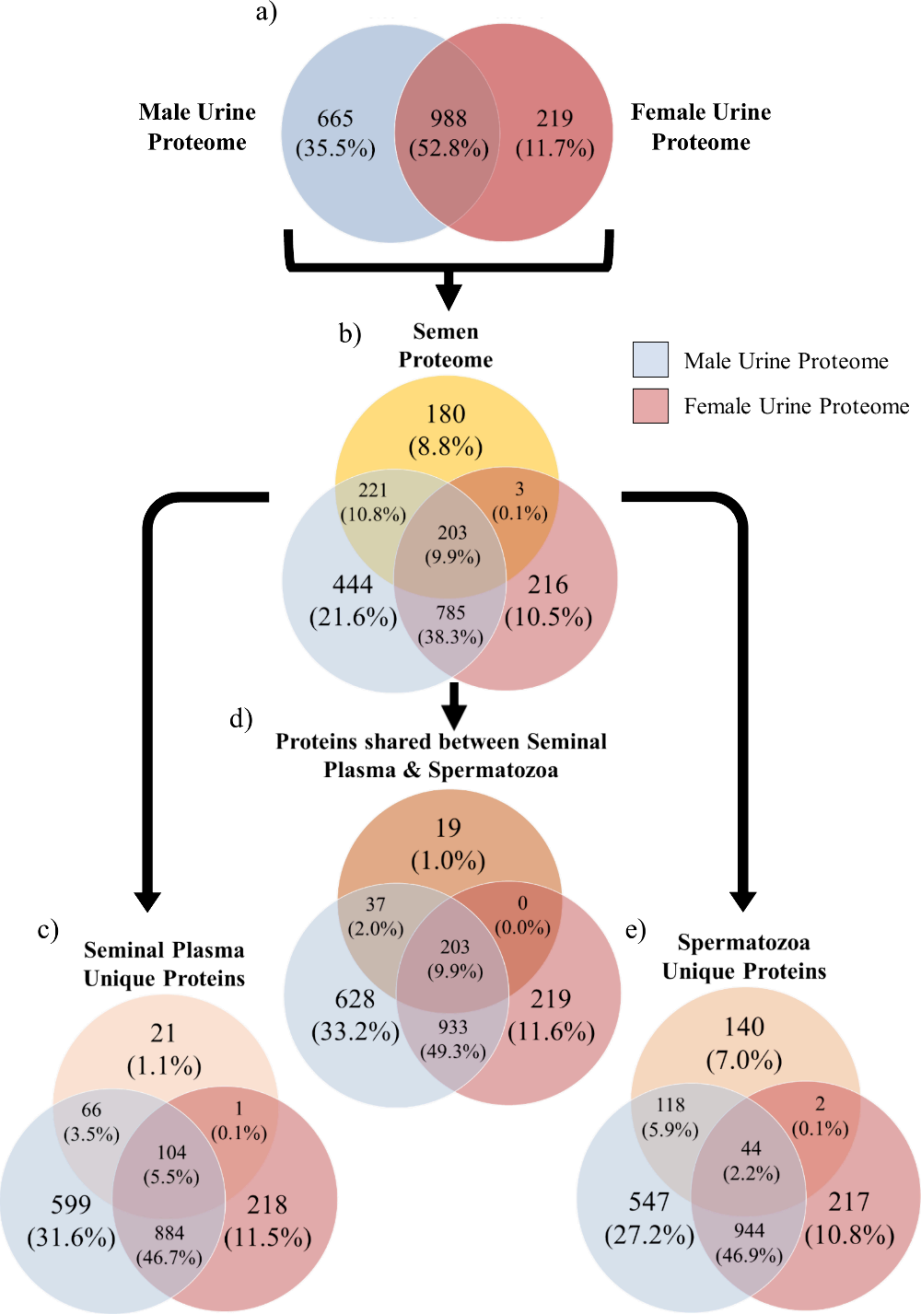
(a) Venn diagram of the number of proteins identified within the male (blue) and female (pink) bottlenose dolphin urine proteomes. (b) Venn diagram of male and female urine proteomes with the semen proteome obtained from the study by Fuentes-Albero et al.^25^ The semen proteome was categorized into three groups: (c) proteins unique to seminal plasma, (d) proteins unique to spermatozoa, and (e) proteins shared between seminal plasma and spermatozoa. The absolute numbers represent the count of identified proteins that are either unique to or shared between the groups. The percentages indicate the number of identified proteins relative to the total number of proteins identified within both the urine and semen proteomes. Venn diagrams are not scaled to size.

### Presence of Putative Antimicrobial Proteins

Several homologous urinary proteins from other mammals, known for their antimicrobial effects against both Gram-positive and Gram-negative bacteria,^35–40^ were identified in the bottlenose dolphin urine proteome. Among the most abundant urine proteins, two putative antimicrobial proteins were found in bottlenose dolphin urine: CAMP (mean rank: 9) and LYZ (mean rank: 21), together constituting 2.66% of their urine protein composition (Figure 2). California sea lion urine contained five antimicrobial proteins, comprising 19% of the urine protein composition. These proteins include ribonuclease A family member k6 (RNASE6; mean rank: 3), LYZ (mean rank: 4), resistin (RTN; mean rank: 12), defensin beta 1 (DEFB1; mean rank: 16), and CAMP (mean rank: 20) (Table S3). California sea lions exhibit a higher fractional abundance of four putative antimicrobial proteins compared to bottlenose dolphins (*p*_*adj*_ < 0.05, Mann–Whitney U test, Benjamini-Hochberg corrected, Figure 9). However, the fractional abundance of CAMP did not differ significantly (*p*_*adj*_ > 0.05, Mann–Whitney U test, Benjamini-Hochberg corrected) between bottlenose dolphins and California sea lions.

**Figure 9:**
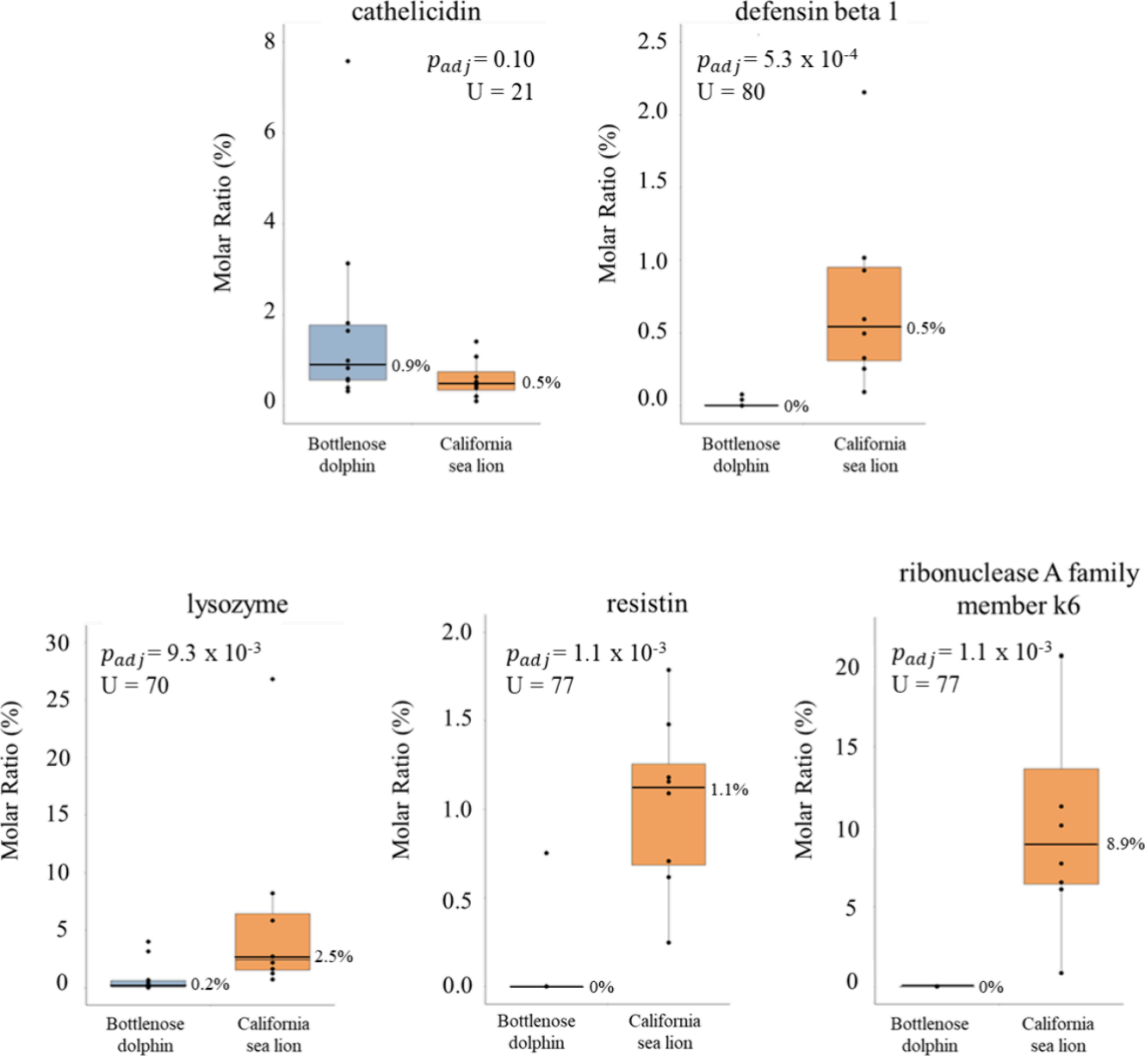
Boxplots of protein molar ratio (%) of antimicrobial proteins identified within the top 30 abundant urine proteins for each species, including bottlenose dolphins (*N* = 10) and California sea lions (*N* = 8). Individual urine samples for each species are represented by dots on the graphs. The box represents the interquartile range (IQR), with the median marked by the horizontal line inside the box, with corresponding value on the right side of the box. Whiskers extend to the minimum and maximum values within 1.5 times the IQR. Four proteins varied significantly between bottlenose dolphins and California sea lions, including defensin beta 1, lysozyme, resistin, and ribonuclease A family member k6 as evidenced by the Mann–Whitney U tests with Benjamini Hochberg adjustment (*p*_*adj*_ < 0.05 and U values plotted in figure for each protein). Conversely, no significant difference of fractional abundance was observed for cathelicidin between bottlenose dolphins and California sea lions (median: 0.91% vs 0.49%, respectively, *U* = 21, *p*_*adj*_ > 0.05).

### Urinary Proteins Markers of AKI & Kidney Stone Formation

In total, 27 urinary proteins, recognized as markers of AKI based on reports from human and veterinary medical research articles,^2,8,41^ were identified in bottlenose dolphin urine (Figure 10; Table S4). Three urine proteins: NGAL, OLM4, and SLC9A3, were present in bottlenose dolphin urine but not in nondiseased California sea lion urine, although NGAL was identified in the diseased California sea lion urine.^8^ Furthermore, several putative renal injury markers were not found in bottlenose dolphins, including CTGF, IL18, CHI3L1, CRP, CYR61, FABP2, FABP4, FABP5, GSTA3, HAVCR1 (alternative symbol: KIM1), RBP2, TFF3, TIMP1, TNFRSF12A, VCAM1.

**Figure 10:**
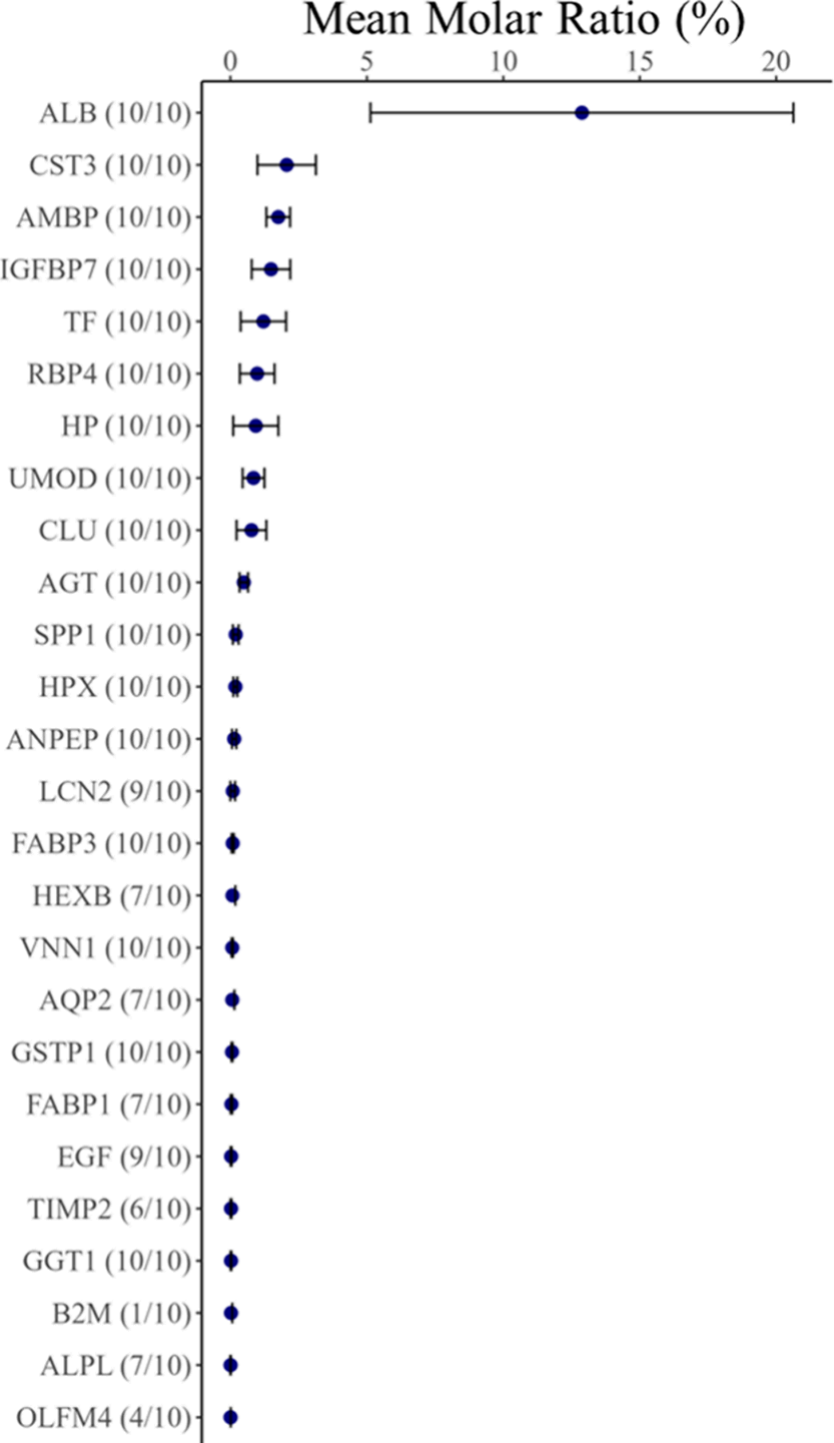
Mean molar ratio (%) of 26 identified urine proteins detected in bottlenose dolphin urine, implicated as acute kidney injury markers in human and domestic veterinary medicine research, determined using normalized emPAI values. The numbers next to the HGNC-approved gene symbols for each protein represent the number of individuals, out of 10, who excreted that protein.

A compilation of urinary proteins homologous to human inhibitors and promoters of kidney stone formation, particularly for calcium oxalate, was compiled to determine their presence or absence in the bottlenose dolphin urine proteome (Table S5). Among these proteins, five out of six identified urine proteins, including UMOD, ALB, LYZ, MPO, and LTF, that promote the formation of kidney stones in humans were found in the bottlenose urine proteome. Eight out of 11 urine proteins, including UMOD, S100A6, S100A12, SPP1, ALB, F2, AMBP, FN1, and TF, known to inhibit kidney formation in humans, were detected in bottlenose dolphin urine. However, three inhibitory proteins were not detected in bottlenose dolphin urine, including REG1A, PCYT1B, and TFF1. One promotive protein, HIST1H1B, was also not detected in the bottlenose dolphin urine.

## Discussion

This study explored the urine proteome of free-ranging bottlenose dolphins and identified putative antimicrobial proteins, AKI markers, and proteins related to kidney stone formation/inhibition. Examining how the bottlenose dolphin urine proteome compares to that of other mammals provides a deeper understanding of the similarities and differences between diving and terrestrial mammals. The consistent identification of common proteins such as ALB, UMOD, AMBP and haptoglobin (HP), underscores the conservation of abundance for certain urinary proteins among diverse mammalian taxa.^1,8,32,33^ The predominant presence of ALB in the bottlenose dolphin urine likely reflects its high plasma concentration, a pattern also observed in humans, California sea lions, and dogs.^1,8,32,42,43^ In contrast, the urine proteome of rodents is dominated by major urinary proteins, specifically α2u-globulins,^44^ while cauxin is the most abundant urinary protein in cats.^3^ Additionally, while UMOD is the second most abundant protein in the urine of dogs and humans,^1,32^ it ranks lower in bottlenose dolphins, occupying the 22nd position in relative abundance. Similar patterns of reduced UMOD abundance have been observed in other mammals, including California sea lions, giraffes, and Bactrian camels.^8,12,13^ These observations suggest that phylogenetic differences exist in the high abundance of urine proteins and that generalizations about urine protein composition across mammalian taxa should be met with skepticism.

In healthy animals, the amount of urine protein should be low,^42^ reflecting an intact glomerular filtration barrier that selectively permits the passage of low molecular weight proteins. While these low molecular weight proteins are largely reabsorbed at the proximal tubule, some are to be excreted in the urine, whereas high molecular weight proteins in the urine are likely to be shed from the tubular cells of the kidney and epithelium of the bladder. The resulting distribution closely resembles that observed in other mammals, including California sea lions, giraffes, humans, and dogs, where most urinary proteins fall within the 10 kDa to 70 kDa range, with some exceeding 70 kDa.^1,13,45,46^ Glomerular filtration is primarily governed by molecular size and charge, with ALB (69 kDa) often serving as a functional threshold for filtration.^47,48^ In bottlenose dolphins, 66% of plasma and 61% of serum proteins fall below this threshold, a distribution similar to that observed in humans, where more than 50% of plasma proteins are under 69 kDa.^49^ Although larger proteins are generally restricted by the glomerular filtration barrier, small amounts still can pass through or be secreted and shed in the nephron and urinary tract.^50,51^ One limitation of this analysis is the assumption that all proteins remain intact and unmodified. Post-translational modifications or proteolytic degradation could alter protein molecular weights.^52^ Despite this consideration, the overall size distribution of urinary proteins in bottlenose dolphins does not appear to vary drastically compared to other mammals.

### Sex-Specific Variations

Despite genetic differences between the two Gulf of Mexico sites,^53,54^ PCA analysis of urine proteins did not reveal site-based clustering; the same was observed for sexes, which presents a limitation to this study. However, CST11 was an unexpected protein that emerged as the third most abundant protein in the bottlenose dolphin urine proteome. This finding appears to be driven by its exclusive presence in male individuals in this study, likely due to its known role as the predominant component of bottlenose seminal plasma.^25^ In humans, CST11 is similarly found in the epididymis and sperm.^34^ The protein was detected in four of five male bottlenose dolphins, suggesting that it enters the urine through the reproductive tract disproportionately influencing the male urine proteome. This effect was particularly evident in two individuals (F292 and YR0), where sperm was observed microscopically in the urine, leading to a 54% higher number of identified proteins compared to males without sperm. The contamination of semen in urine samples highlights a key limitation in bottlenose dolphin urine collection methods that do not exclude sperm, whereas techniques such as cystocentesis or Foley catheterization used in other species, such as California sea lions, dogs, and humans^1,8,32^ may be less affected. The absence of CST11 in the urine of female bottlenose dolphins and the high abundance of CST11 in male bottlenose dolphin samples that contained sperm, suggests that CST11 could serve as a useful marker for detecting semen contamination in bottlenose dolphin urine. This suggests that variation in the urine proteome is primarily driven by sex-specific factors, such as CST11, rather than other factors (e.g., population, life stage, environment). Additionally, proteomic data of male bottlenose dolphin urine samples may not exclusively reflect a urine-based proteome, which is an important consideration for interpreting proteomic data.

### Presence of Putative Antimicrobial Proteins

A systematic examination of antimicrobial proteins in urine has not been conducted to date, but several studies have mentioned the presence of these proteins in the urine of various mammals.^8,12,13^ The comparatively high rank abundance of putative antimicrobial proteins to the total protein composition in the urine of bottlenose dolphins and California sea lions highlights a phenotypic congruence, despite the lack of a recent evolutionary relationship between the species. In bottlenose dolphin urine, LYZ and CAMP emerge as prominent antimicrobial proteins, while California sea lion urine contains a broader array, including five putative antimicrobial proteins. These proteins are known to be effective against both Gram-negative and Gram-positive bacteria by directly killing them, suggesting that both species may have evolved similar defense mechanisms.^35–40,55,56^ It is possible that the presence of antimicrobial proteins in bottlenose dolphin urine could be a consequence of phylogeny rather than a unique adaptation shaped by evolution. For instance, terrestrial mammals such as giraffes and Bactrian camels, which share a common ancestor with bottlenose dolphins,^57,58^ were reported to excrete LYZ and CAMP in their urine in high abundance.^12,13^ This suggests that these protective proteins may have been conserved across evolutionary time due to selective pressures on a common ancestor of artiodactyls. However, the exact roles and evolutionary significance of these antimicrobial proteins in urine remain to be fully elucidated. The higher diversity seen among these proteins in California sea lions compared to bottlenose dolphins could be attributed to evolutionary divergence or differing microbial exposures, such as those encountered through beach microbes, temperature fluctuations, and social interactions, which increase the risk of bacterial diseases like leptospirosis.^59,60^ These factors may have driven the higher abundance of antimicrobial proteins in California sea lions compared to bottlenose dolphins, reflecting species-specific responses to environmental pressures. Importantly, no studies have demonstrated antimicrobial activity in marine mammal urine and the presence of these proteins should not imply biological activity.

### Presence of Markers of AKI and Kidney Stone Formation

Although none of the bottlenose dolphins in this study exhibited evidence of renal injury based on serum chemistry data and light microscopic evaluation of urine sediment, their urine samples revealed 26 proteins known to serve as urinary biomarkers for assessing AKI in humans (Table S4). Interestingly, two well-established AKI biomarkers, KIM-1 and IL18, were absent from the bottlenose dolphin urine despite their recognized roles in ischemia-reperfusion injury and AKI detection in humans.^41,61–64^ However, the presence of other candidates, such as NGAL, suggests alternative biomarkers may be more relevant for assessing renal health in bottlenose dolphins. NGAL, a 23 kDa protein and an early biomarker for ischemic, septic, or nephrotoxic kidney injury^65^ was readily detectable in bottlenose dolphins of this study; whereas it was not readily detectable in California sea lions with normal renal function,^8^ suggesting NGAL concentrations may be higher in apparently healthy bottlenose dolphins. This protein is typically produced at low concentrations by renal epithelial cells but is rapidly upregulated following renal injury in humans.^41,66^ Although no evidence of renal injury was identified by light microscopic evaluation of urine sediment (e.g., casts), it is notable that exercise and stress from capture may have altered the urinary proteome as reported in urine of humans.^67^ Beyond its role as a biomarker, NGAL has been shown to activate autophagy and reduce renal tubular cell death during ischemia-reperfusion events, potentially serving a renoprotective function.^68^ Interestingly, inhibitory kappaB kinase has been implicated in the regulation of NGAL in rats through the promotion of NF-kappa B activity suggesting perhaps in bottlenose dolphins the basal state of inhibitory kappaB kinase is more active compared to California sea lions or other terrestrial mammals.^69^ Whether NGAL excretion increases or decreases in bottlenose dolphins with renal injury remains unknown. Regardless of its exact role, the detection of NGAL in bottlenose dolphins means that it can be assessed as a candidate biomarker for renal injury. The findings from this study can serve as a resource for future biomarker studies of renal health, facilitating protein measurements through parallel reaction monitoring approaches similar to those published for bottlenose dolphins with metabolic disease.^70,71^

Bottlenose dolphins under human care are prone to developing ammonium urate kidney stones and show a higher susceptibility to insulin resistance and metabolic syndrome compared to free-ranging bottlenose dolphins.^16,17,72,73^ In humans, numerous urinary proteins have been implicated in modulating stone growth or inhibition, particularly calcium oxalate stones (Table S5). To determine whether these proteins are present in bottlenose dolphin urine, we recognized 11 proteins that influence stone inhibition or growth. From published literature, six proteins exhibit inhibitory properties, three proteins exhibit promotive effects, and two proteins modulated growth with context-dependent roles (Table S5). Although there was no clinical concern for kidney stones in the bottlenose dolphins in this study, the consistent presence of stone inhibitors suggests physiological similarities with other mammals. However, a notable absence in bottlenose dolphin urine was TFF1, a potent inhibitor of stones in humans.^74–76^ Inspection of the bottlenose dolphin genome revealed an absence of this gene. Whether the absence of TFF1 is relevant to the higher incidence (35%) of ammonium urate stones observed in managed groups^16^ remains to be determined. This also raises the question of whether bottlenose dolphins rely on alternative proteins or physiological adaptations to prevent stone formation. Adding to the complexity, proteins like UMOD exhibit dual functionality, promoting stone aggregation under high calcium concentrations or inhibiting growth under alkaline conditions.^77,78^ However, because bottlenose dolphin kidney stone cases are predominantly reported as ammonium urate rather than calcium oxalate, the precise role of these proteins in stone formation remains unclear. Further comparative proteomic studies of managed bottlenose dolphins with and without ammonium urate stones, alongside free-ranging bottlenose dolphins, are necessary to uncover potential protective mechanisms and better understand the factors influencing nephrolithiasis in these species.^79^

### Limitations

This study is based on a small sample size of ten bottlenose dolphins, which may limit the generalizability of the findings. The sampling constraints led to an unequal site distribution, and bottlenose dolphins with known renal insufficiency were not included, further restricting the scope of the analysis. In addition, there was a high variability in the number of proteins detected across individuals, which could arise from biological differences or interteam variations in the sampling protocols (e.g., sample handling and processing). In humans, factors such as protein concentration, pH, age, health, diet, stress, exercise, and proteolysis can influence urine composition.^50,80–82^ Similar factors are likely to contribute to these variabilities in bottlenose dolphin urine proteome, making it challenging to discern differences between sexes and sites. For instance, the absence of distinct clustering in urine proteomes by sampling sites contrasts with genetic studies of bottlenose dolphins from Barataria Bay and Sarasota Bay, which show site differences.^53,54^

Additionally, limited comparative analyses of urine proteomes in other mammalian species, especially artiodactyls like cows and horses, hinder direct comparisons. While some data sets exist (e.g., adult cow urine proteomes^83^), attempts to replicate comparable search strategies were unsuccessful. Urine proteome data for species like giraffes^13^ and Bactrian camels^12^ are not publicly available, leaving only published literature for cross-species comparisons. Establishing standardized protocols for assessing urine proteins across species and making data available using universally accepted file formats will improve the reliability of comparisons between our data and those from other mammals.

## Conclusions

This study presents the first comprehensive analysis of urine proteins in bottlenose dolphins, offering insights into the species and differences and similarities compared to other mammals, including California sea lions, dogs, giraffes, Bactrian camels, and humans. A key observation was semen contamination in male bottlenose dolphin urine, which elevates protein representation compared to females. The high relative abundance of antimicrobial proteins, such as lysozyme and cathelicidin, is consistent with that found in another marine mammal suggesting a potential role in protecting bottlenose dolphins from urinary tract infections. Additionally, this study produced a list of proteins that will allow researchers to include or exclude candidates for protein markers in future studies investigating renal injury and disease based on presence or absence, thereby prioritizing protein markers that are known to be present. This data set, alongside traditional markers such as SCr and BUN, has utility to identify biomarkers useful in the early detection of renal disease in bottlenose dolphins.

## Supplementary Material



## Data Availability

The proteomics data obtained through mass spectrometry have been archived in the ProteomeXchange Consortium (http://proteomecentral.proteomexchange.org). The data set is identified as PXD054283 and is available through the PRIDE partner repository.

## Notes

(Table S1) Urine and serum chemistry data for individual bottlenose dolphins; (Table S2) molar ratio (%) as determined by normalized exponentially modified protein abundance index (emPAI) for each bottlenose dolphin; (Table S3) molar ratio (%) as determined by exponentially modified protein abundance index (emPAI) for nondiseased California sea lions (control); (Table S4) identification and molar ratio (%) of 27 reported markers of kidney injury in bottlenose dolphins; (Table S5) list of urinary proteins implicated in the modulation (inhibitors or promoters) of kidney stone formation, particularly calcium oxalate stones, based on findings from human medicine studies (XLSX)

## Abbreviations

AKI: acute kidney injury
BUN: blood urea nitrogen
SCr: serum creatinine
